# Draft Genome of the Entomopathogenic Fungus Metarhizium robertsii DSM 1490

**DOI:** 10.1128/mra.01267-22

**Published:** 2023-04-05

**Authors:** Tilottama Mazumdar, Alexander Bartholomäus, Dino P. McMahon

**Affiliations:** a Institut für Biologie, Freie Universität Berlin, Berlin, Germany; b Department for Materials and Environment, BAM Federal Institute for Materials Research and Testing, Berlin, Germany; c GFZ German Research Centre for Geosciences, Section Geomicrobiology, Potsdam, Germany; Vanderbilt University

## Abstract

Metarhizium robertsii DSM 1490 is a generalist entomopathogenic fungus. The mechanisms of pathogenesis of such fungi in insects like termites are not completely understood. Here, we report the draft genome sequence, as sequenced on the Oxford Nanopore platform. The genome has a GC% of 47.82 and a size of 45,688,865 bp.

## ANNOUNCEMENT

The fungal entomopathogen Metarhizium robertsii is a generalist obligate-killing entomopathogenic fungus capable of infecting a number of insects, including termites, where it is detected in soils in close proximity to colonies (1). The mechanisms of pathogenesis of generalist Metarhizium entomopathogens remain interesting and ongoing areas of research. For further exploration of the virulence mechanisms of such fungi, for example, via gene knockout, a reliable genome sequence is required.

*Metarhizium robertsii* DSM 1490 was obtained from the German Collection of Microorganisms and Cell Cultures GmbH (DSMZ). Until 2022, this strain was classified as Metarhizium anisopliae by the German Collection of Microorganisms and Cell Cultures GmbH (DSMZ) (https://www.dsmz.de/collection/catalogue/details/culture/DSM-1490). The strain has been used to successfully infect termites (2). Conidiospores were grown on potato dextrose agar (PDA). The spores from the PDA surface were scraped off using sterile swabs and 0.05% Tween, followed by centrifugation (2,800 × *g*, 15 min) and resuspension in 0.05% Tween. This washing step was repeated two further times, before spores were counted in a hemocytometer. A total of 1 × 10^8^ spores were inoculated in liquid modified medium (40 g/L yeast extract, 80 g/L glucose, 0.1% Tween 80) and allowed to grow at 25°C, 290 rpm, for 3 days. The mycelial mass was filtered from the blastospores using double-layered sterile Miracloth, and DNA was extracted from 500 mg of mycelia. Tissue homogenization was carried out with 2-mm beads in a tissue lyser (MP Biomedicals, FastPrep-24), after adding 500 μL lysis buffer (10 mM Tris HCl, 1 mM EDTA, 100 mM NaCl, and 2% SDS). After proteinase K treatment (20 μL, 56°C for 3 h) and isolation of the nucleic acid fragment by phenol-chloroform-isoamyl alcohol phase separation (25:24:1), the resulting nucleic acid underwent RNase A treatment (2 μL, 37°C for 1 h). The quality of the DNA was assessed using a NanoDrop spectrophotometer, and the size of the fragment was checked on a 1.5% agarose gel to confirm the presence of large DNA fragments (>10,000 bp).

DNA was purified using AMPure XP beads (Beckman Coulter, Pasadena, CA), followed by library preparation using the SQK-LSK110 kit (Oxford Nanopore Technologies [ONT], Oxford, UK), according to the manufacturer’s instructions. The DNA was sequenced on a MinION sequencer and the MinKNOW v22.05.5 tool (ONT), using a single FLO-MIN106 (R9.4.1) flow cell for 72 h. Reads were base called and demultiplexed using Guppy v6.1.5 (ONT) at superhigh-accuracy mode, resulting in 3.8 million reads with an estimated *N*_50_ of 8.57 kb. The high-quality reads (classified pass by Guppy) were assembled using Flye v2.9 (3), with default parameters. Medaka v1.5.0 (4) was used to polish the draft assembly. Assembly metrics were calculated using QUAST v5.0.2 (5) (Table 1). The assembly resulted in 29 contigs, with an *N*_50_ of 4.6 million bp, a total size of 45,688,865 bp, and a GC% of 47.82. The genome completeness was assessed using BUSCO v5.1.2 (6) (parameter: lineage ascomycota_odb10), showing a completeness score of 96.8% (complete BUSCOs [C], 96.8%; missing BUSCOs [M], 2.2%; total BUSCO groups searched [*n*], 1,706).

**TABLE 1:** QUAST summary output of assembly metrics

Metric	Assembly statistic
No. of contigs of size	
≥0 bp	29
≥10,000 bp	29
≥25,000 bp	27
≥50,000 bp	25
Total length (bp) of contigs of size	
≥0 bp	45,688,865
≥10,000 bp	45,688,865
≥25,000 bp	45,661,755
≥50,000 bp	45,593,025
Largest contig (bp)	7,469,598
GC (%)	47.82
*N*_50_	4,618,093
*N*_75_	3,381,972
*L*_50_	4
*L*_75_	8
No. of N’s per 100 kbp	0.00

### Data availability.

The draft genome of *Metarhizium robertsii* DSM 1490 has been deposited at NCBI with accession number SAMN31743403 (GenBank accession number JAPPVM000000000.1), and the raw reads have been deposited at NCBI with the SRA identifier (ID) SRX18385371 and BioProject number PRJNA899806. The strain is available at the German Collection of Microorganisms and Cell Cultures (DSM 1490).
